# Use of proton pump inhibitors are associated with higher mortality in hospitalized patients with COVID-19

**DOI:** 10.7189/jogh.12.05005

**Published:** 2022-02-19

**Authors:** Shengyong Wu, Zhichao Jin, Chi Peng, Dongdong Li, Yi Cheng, Ronghui Zhu, Jia He, Cheng Wu

**Affiliations:** Department of Military Health Statistics, Naval Medical University, Shanghai, China

## Abstract

**Background:**

The adverse effects of proton pump inhibitors (PPIs) on pneumonia have been well reported. However, the relationship between the use of PPIs and the adverse outcomes of coronavirus disease 2019 (COVID-19) is currently inconclusive. In this study, we aimed to explore the relationship between the use of PPIs and the in-hospital mortality among patients who were laboratory-confirmed SARS-CoV-2.

**Methods:**

Data was derived from 2 hospitals which both were the first batch of SARS-CoV-2 specialist hospitals with four types of sensitivity analyses. This cohort included 4634 patients older than 18 years who were laboratory-confirmed SARS-CoV-2. Endpoints were death in hospital (primary) and the recovery of COVID-19 (secondary: the time of COVID-19 nucleic acid testing turning negative).

**Results:**

In the entire cohort, there were 3588 non-users, 399 ≤ 0.5 defined daily dose (DDD) PPIs users, 483 1 DDD users, and 164 ≥ 1.5 DDD users. The multivariate logistic regression analysis (odds ratio (OR) = 3.63, 95% confidence interval (CI) = 1.83-7.23, *P* = 0.0002) and four types of sensitivity analyses showed higher mortality in patients using PPIs during hospitalization, while the relationship between different PPIs dosages and the hospital mortality remained insignificant. Usage of the PPIs significantly prolongs the time of COVID-19 nucleic acid testing turning negative.

**Conclusions:**

The use of PPIs may increase the risk of in-hospital death of patients who were laboratory-confirmed SARS-CoV-2, which means that physicians may need to re-evaluate the benefit-risk assessment of the use of PPIs during the COVID-19 pandemic.

The novel coronavirus severe acute respiratory syndrome coronavirus 2 (SARS-CoV-2), which has spread worldwide, has been reported first in December, 2019 [1]. By the end of September 14, the COVID-19 pandemic has caused more than 225 million people to be confirmed and more than 4 million people deaths [2]. In-hospital mortality rates have varied widely among different nations and therapeutic regimens, and adverse drug reactions are still being identified [3-6].

Proton pump inhibitors (PPIs) are the most potent inhibitors of gastric acid secretion in the clinical use of drugs [7]. Like the significant effect in digestive diseases, in 2015, PPIs were in the top 10 related drug expenditures in the USA [8]. Furthermore, PPIs become drugs of choice for stress ulcer prevention in critically ill patients [9]. However, in 2014 an observational study of 35 312 patients initiated by Allen et al. [10] reported that PPIs were associated with a higher risk of gastrointestinal tract hemorrhage (odds ratio (OR) = 2.24, 95% confidence interval (CI) = 1.81-2.76), pneumonia (OR = 1.20, 95% CI = 1.03-1.41), and Clostridium difficile infection (OR = 1.29, 95% CI = 1.04-1.64) than histamine-2 receptor antagonists. Moreover, a survey raised by Sameer et al. [11], who invited 799 internists in the USA, shows that 70% of the respondents were somewhat/very concerned about PPIs adverse effects (AEs).

As a novel severe infectious disease, COVID-19 pandemic was suggested that 5%-20% of patients develop a critical illness characterized primarily by acute respiratory distress syndrome [12-15], which implies that the prevention of stress ulcers is a question which doctors must face. As the first batch of COVID-19 pandemic specialist hospitals, Huo Shen Shan Hospital and Guang Gu District of the Maternal and Child Health Hospital of Hubei Province, Wuhan, China, collected previous first-hand data of the patients and effectiveness of drug treatment. Here, we characterize PPIs` usage, epidemiology, clinical course, and risk factors for in-hospital mortality among a cohort of adults with COVID-19 admitted to that hospital as mentioned above during the four months of the city’s outbreak.

## METHODS

### Data source

Data were obtained from the de-identified patient database of Huo Shen Shan Hospital and Guang Gu District of the Maternal and Child Health Hospital of Hubei Province, Wuhan, China. This database provided data of all individuals diagnosed with laboratory-confirmed COVID-19 pandemic and admitted by the hospital as mentioned above (n = 4804). The China Government provided free medical care for all patients with COVID-19 during the pandemic. Therefore, the acquired information including personal information, medication administration records, nursing records, laboratory results, admitting diagnosis, and discharge diagnosis. All records we use for this research were anonymized to ensure confidentiality.

### Ethical considerations

This study was conducted by the amended Declaration of Helsinki. Ethical approval was granted by the Ethical Committee of Huo Shen Shan Hospital (HSSLL030) and Naval Medical University. Due to the emergency and risk of contagion, written informed consent was not required.

### Study population

**Figure 1:**
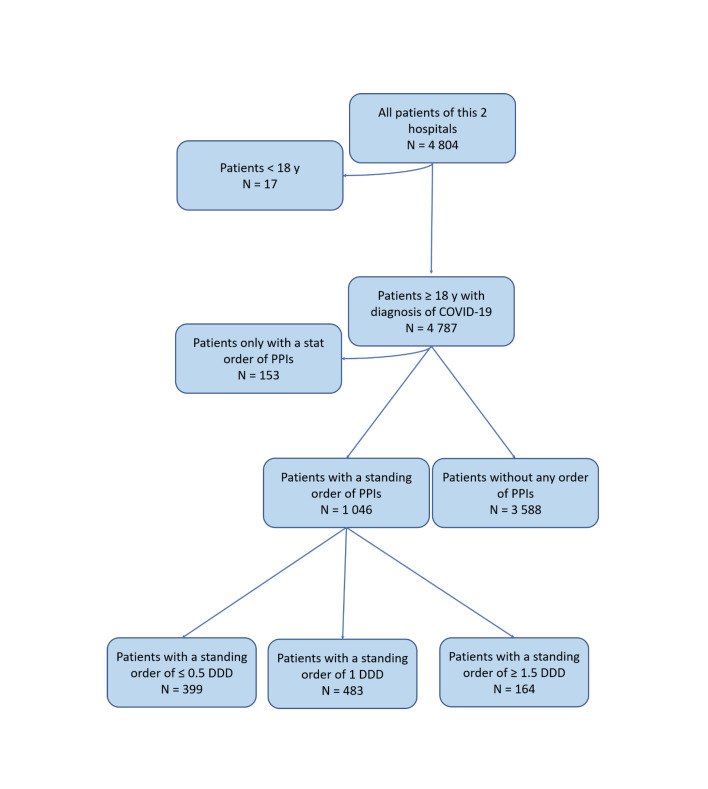
Flow diagram of target population.

The date of each patient admitted to the hospital was defined as the entry date of the cohort. Among the total of 4 804 patients who confirmed COVID-19, patients were excluded if they were younger than 18 years old (n = 17) or they just had a stat order of PPIs (n = 153). The final sample which confirmed COVID-19 comprised 4 634 individuals, of whom 399 have a standing order of ≤0.5 defined daily dose (DDD) PPIs, 483 have a standing order of ≤1 defined daily dose (DDD) PPIs, and 164 have a standing order of ≥1.5 defined daily dose (DDD) PPIs. (**Figure 1**).

The laboratory confirmation of SARS-CoV-2 infection was defined as twice positive result consecutively of real-time reverse transcriptase PCR assay of nasal and pharyngeal swabs, which will take the third test if the second test is negative. The patient's medical insurance types were taken from the home page of the medical records. The breathing rate, admission temperature, admission systolic blood pressure, admission diastolic blood pressure, and pulse rate were collected from the nursing records. The laboratory test results, past medical history, diagnosis, and medication use were all confirmed by medical records systems.

### Exposure

We identified all PPIs (omeprazole, rabeprazole, lansoprazole) prescribed by standing order during hospitalization. Defined daily dose (DDD) was used as the unit for measuring a prescribed amount of drug. Based on the ratio of the actual daily dose of the patient to the DDD, the following three groups of patients were distinguished for dosing: ≤0.5 DDD, 1 DDD, and ≥1.5 DDD. Non-users were defined as patients who had never received stat order or standing order of PPIs during hospitalization.

### Outcomes

The primary outcome was in-hospital mortality, with additional secondary outcome of the time of COVID-19 nucleic acid testing turning negative which was tested daily.

### Missing variables

Values were missing for <5% of individuals for most variables; however, type of medical insurance and serum globulin were missing for 10.53% and 14.78% of patients (Table S1 in the **Online Supplementary Document**), respectively. Missing data were imputed with medians or dominant category depending on whether they were continuous or categorical information [16,17].

### Statistical analysis

Univariate analysis of the comparisons between two groups was determined by Student’s *t* test or Mann-Whitney U test for normal distributed or non-normal distributed continuous variables, and Pearson Chi-Square test or Mann-Whitney U test for unordered categorical variable or ordinal categorical variable. We constructed a univariate logistic model for preliminary screening first. Then the variables from the univariate logistic regression that had a *P*-value less than 0.15 were used to constructed multivariate logistic regression analysis (Model 1) to identification of independent risk factors (stepwise, sls = 0.10, sle = 0.05).

Patient variables included in the analysis were age, sex, type of medical insurance, severity of illness, admission temperature, admission systolic blood pressure, admission diastolic blood pressure, pulse rate, peripheral red blood cell count, peripheral white blood cell count, absolute mononuclear leukocyte count, absolute neutrophil count, absolute lymphocyte count, absolute thrombocyte count, glutamic-pyruvic transaminase, glutamic-oxalacetic transaminase, serum creatinine, serum albumin, serum globulin, total bilirubin (TBil), alkaline phosphatase, admission oxyhemoglobin saturation, hypertension, diabetes, malignant tumor, stroke history, chronic obstructive pulmonary disease (COPD), coronary heart disease history, digestive system disease, antiviral treatment, antibiotic therapy, antifungal therapy, immunotherapy, hormonotherapy and transfer to intensive care unit (ICU) (Table S2 in the **Online Supplementary Document**). Due to the small proportion of patients with some indicators above or below the range of normal values, normal or abnormal is used as an evaluation indicator.

Survival curve of COVID-19 nucleic acid testing turning negative time between the two groups using PPIs and without PPIs was estimated with the Kaplan-Meier method and compared by log-rank test in the cohort matching by propensity score matching (PSM). Univariate Cox regression analyses were carried out to estimate the hazard ratio (HR) and 95% confidence interval (CI).

All tests were two-tailed, and *P* < 0.05 was considered significant unless otherwise specified. Statistical analysis was performed using SAS version 9.4 (SAS Institute Inc) and R version 4.0.4 (R Foundation for Statistical Computing, Vienna, Austria).

### Sensitivity analysis

Four sensitivity analyses were performed to assess the robustness of our findings. One multivariate logistic regression analysis was constructed using the same variables as model 2, replacing the use of PPIs or not with different doses of PPIs, in order to reduce the effects of PPIs doses.

For the sake of decreasing the effects of confounding and likelihood of selection bias, we performed secondary analyses using propensity score matching (Model 3) and propensity score weighting (Model 4). The propensity score was performed using a logistic regression model with adjustment for the same covariables as model 1 (Table S3 in the **Online Supplementary Document**).

Propensity score matching (PSM) was performed using the ‘greedy nearest-neighbor’ algorithm and calculated the predicted probability of users of PPIs vs non-users among all patients who were confirmed of SARS-CoV-2 infection with 1:2 matching with a caliper distance of less than 0.05. Moreover, propensity score weighting (PSW) was performed by the way of average treatment effect for the treated (ATT). After weighting based on the IPTW method, all covariables are also included in the multi-variates model. Adequacy matching and weighting for no significant imbalance of each baseline covariate were assessed by standardized mean differences (SMDs) [18].

One multivariate logistic model (Model 5) was still constructed using the data without imputing the missing data as one way of sensitivity analysis.

### Patient and public involvement

As most patients are in critical condition, no patients were involved in designing or conducting the research. Furthermore, due to the sudden outbreak of the epidemic, no patients were asked to provide advice on analysis or dispose of the results. There are no plans to involve patients or the relevant patient community in the dissemination of study findings at present.

## RESULTS

### Descriptive overview

Among the total of 4634 patients who were confirmed COVID-19, we identified 3588 without the use of PPIs, 399 ≤ 0.5 defined daily dose (DDD) PPIs users, 483 1 DDD PPIs users, and 164 ≥ 1.5 DDD PPIs users administration in the whole unmatched cohort. The baseline characteristics of the entire cohort and two groups of individuals are showed in Table S2 in the **Online Supplementary Document****,** 2478 (53.47%) of the individuals were aged between 41 and 65 years in the entire cohort, and 2222 (47.95%) were males. Individuals using PPIs were likely to be older, with high rates of relevant medical history and abnormal laboratory findings (Table S2 in **the** **Online Supplementary Document**).

### In-hospital mortality risks for the use of PPIs in hospitalized patients

Overall, in-hospital mortality was 1.99%. There was significantly higher mortality (Table S2 in the **Online Supplementary Document**) in patients using PPIs during hospitalization compared with patients without use of PPIs (6.97% (73/1047) vs 0.53% (19/3604), *P* < 0.0001), and the use of PPIs was associated with an increased risk of death in multivariate logistic analysis (Table S3 in the **Online Supplementary Document**, OR = 3.63, 95% CI = 1.83-7.23, *P* = 0.0002).

In the sensitivity analysis, model 2 (Table S3 in the **Online Supplementary Document**) shows that the risk of mortality was significant in all 3 doses of PPIs, ≤0.5 DDD (OR = 2.97, 95% CI = 1.18-7.51, *P* = 0.0214), 1 DDD (OR = 3.59, 95% CI = 1.70-7.59, *P* = 0.0008), and ≥1.5 DDD (OR = 4.95, 95% CI = 1.96-12.47, *P* = 0.0007). However, there were no statistically significant differences in the mortality rates found between the three groups of different PPIs dosage.

In these cohorts, individuals without the use of PPIs (n = 3604) and those with use of PPIs (n = 1047) were matched equally in our propensity score matched cohorts (Table S4 in the **Online Supplementary Document**) and 780 pairs were matched. No significant imbalances in the demographics and clinical characteristics were observed when evaluated using SMD within groups in the propensity matched and weighted cohorts (Table S4, Figure S1 and Figure S2 in the **Online Supplementary Document**; all SMD<0.10). Moreover, in the propensity score matched cohorts (Table S3 in the **Online Supplementary Document**, Model 3), there was significantly higher mortality in individuals using PPIs during hospitalization (OR = 2.95, 95% = CI 1.57-5.52, *P* = 0.0008). As for the propensity score weighted cohorts (Table S3 in the **Online Supplementary Document**, Model 4) which we used a double robust method to reduce bias, multivariate logistic analysis corroborates these results above (OR = 2.97, 95% CI = 1.63-5.42 *P* = 0.0004). The multivariate analysis of the data without missing data imputing (Table S5 in the **Online Supplementary Document****,** Model 5) obtained the same result (OR = 6.35. 95% CI = 2.61-15.43, *P* < 0.0001) as above (Table S5 in the **Online Supplementary Document**).

### The time of COVID-19 nucleic acid testing turning negative risks for the use of PPIs in hospitalized patients

**Figure 2:**
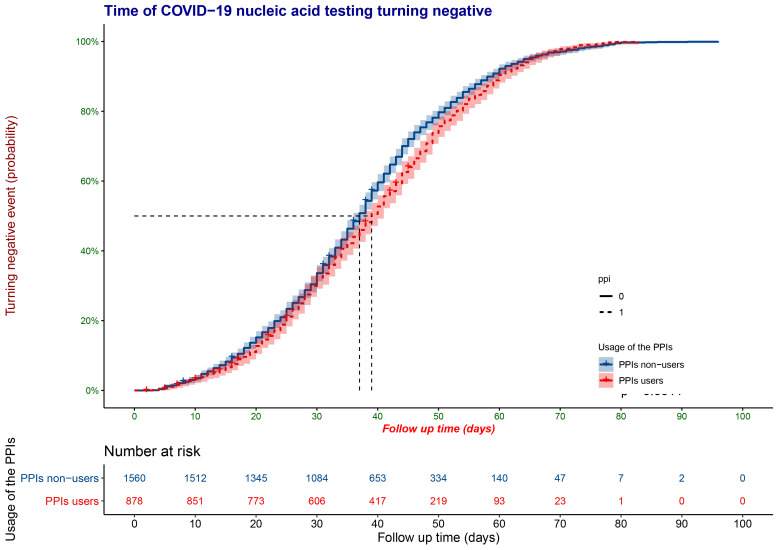
Kaplan-Meier estimates of the COVID-19 nucleic acid testing turning negative event.

The median time of the COVID-19 nucleic acid testing turning negative of all the patients was 38 days (interquartile range = 26-47). In the PSM cohort, the COVID-19 nucleic acid testing turning negative time (**Figure 2**) of patients using PPIs was significantly longer than those without using PPIs (38 vs 37 days, log-rank *P* = 0.0311) and the HR were 0.91 (95% CI = 0.84-0.99).

## DISCUSSION

In this study, we investigated whether the use of PPIs increased the risk of in-hospital mortality among 4 634 patients who were confirmed COVID-19, as well as whether there were longer the time of COVID-19 nucleic acid testing turning negative among all individuals. After the adjustment for covariates and four types of sensitivity analyses, we found that the use of PPIs during hospitalization may increases the risk of in-hospital mortality and longer the time of COVID-19 nucleic acid testing turning negative; however, there were no statistically significant differences in the mortality rates between the two groups of different PPIs dosages.

### Results explanation

Previous studies have confirmed that one of the target cells for infection of the SARS-CoV-2 were respiratory epithelial cells [19]. In multiple studies, the ACE 2 of type 2 alveolar epithelial cells, which are also expressed in intestinal cells, have been highlighted that may serve as a receptor for serve acute respiratory syndrome coronavirus 1 [19-22], which means the gastrointestinal tract may serve as one of the entries [23]. The severe clinical outcomes due to cytokine storm may be causing by the higher viral entry to cells because of the higher expression of ACE-2 [24,25].

Due to the high-level expression of ACE-2 caused by the use of PPIs, [26] patients who use PPIs during hospitalization may suffer high viral loads, which may in turn cause lethal outcomes. Meanwhile, even in the respiratory tract-related illnesses, individuals with the increase of gastric alkalinity caused by PPIs maybe lead to a higher susceptibility for more virus colonization in the stomach, which may lead to the same result of high mortality, too [27]. Monteleone et al. confirmed that patients undergoing anti-cytokine therapy, which may have a protective effect against the cytokine storm, might reduce the risk of severe clinical outcomes of COVID-19 [28]. In this respect, the enhancement to cytokine storm of the patients using PPIs may contribute to poor outcomes of COVID-19. In the research conducted by Zhou et al. about the Middle East respiratory syndrome coronavirus (MERS-CoV), the mice with PPIs treated suffer lethal outcomes, which suggest that the human intestinal tract may be an alternative route for the Infection of MERS-CoV; this result supports our finding between higher mortality in hospitalized and PPIs usage [29].

Previous studies reported that the use of PPIs might be more vulnerable to infectious complications such as the development of ARDS [30]. Furthermore, a meta-analysis conducted by Hariyanto et al. shows that PPIs usage was significantly associated with the mortality during hospitalization (RR = 1.72, 95% CI = 1.02-2.89) of the patients identified COVID-19 [31]. In this respect, the above studies all came to the same conclusion as we did.

### Strengths and limitations

The strengths of our study may include the following points. First, although several studies have been conducted to confirm that the use of PPIs may be associated with an increased risk of SARS-CoV-2 infection [27], there are fewer studies on PPIs use during hospitalization and patient outcomes. We believe that our study provided potential quantitative evidence about the association between the in-hospital mortality of the patients suffer COVID-19 and PPIs use during hospitalization, which may provide reference for doctors to make clinical decisions on the use of medicines.

Followed, as most of the current studies on the relationship between PPIs and COVID-19 are based on demographic information, this study systematically analyzed the relationship between in-hospital mortality, the time of COVID-19 nucleic acid testing turning negative, and PPIs by combining patient demographic information and various clinical indicators, providing some support for future primary research.

Lastly, being the first batch of the COVID-19-specific hospitals in the world, doctors of these two hospitals did not have an in-depth understanding of COVID-19 as a respiratory virulent infectious disease at the beginning of their treatment, which means that their experience and related results also provide a reference for future management of unexpected and unknown respiratory public health events.

However, our study has several limitations. First, due to the suddenness of the outbreak and the critical condition of most of the patients, it was difficult to ask for and obtain information about some patients, such as weight, height, chronic HIV infection, genetic polymorphisms, smoking, alcohol, and so on [1,32-35], However, our study took into account the more important influencing factors, such as the history of malignant tumor, COPD, and so on, which provide a more comprehensive assessment of the patients.

Second, as a clinically important indicator, length of stay (LOS) was not included in our study. Due to different pressures on hospitals to accept new patients at different times, some patients being able to stay in hospital until they recover and are discharged, and some patients being transferred to other hospitals for follow-up treatment, data provider believes that LOS may have had a limited role in this study. To fill this shortcoming, we compared the time of COVID-19 nucleic acid testing turning negative between two groups.

Finally, our finding of COVID-19 in-hospital mortality and PPIs usage were based on the data from the first batch of SARS-CoV-2 specialist hospitals, which means that the treatment options are not the same as the current established treatment options, especially in the use of hormonotherapy. Although we use several methods to balance this deficiency, it may still lead to bias in the estimation of the results.

In summary, though our study is rudimentary, it may offer a new way for exploring the pathogenesis and progression of COVID-19 and related diseases. Furthermore, we hope that more comprehensive studies will be conducted in the future.

## CONCLUSIONS

Notably, PPIs use in COVID-19 patients during hospitalization significantly increased in-hospital mortality, and the time of COVID-19 nucleic acid testing turning negative. However, there was no statistically significant difference in the risk of in-hospital mortality between the doses of PPIs. Given the recent massive global epidemic of COVID-19 and the widespread use of PPIs worldwide, there is an urgent need for health care professionals to revisit the use of PPIs in patients with COVID-19. We hope that this study may contribute to the treatment of patients with COVID-19 and the control of the global outbreak.

## Additional material


Online Supplementary Document

## References

[1] Grasselli G Zangrillo A Zanella A Antonelli M Cabrini L Castelli A Baseline Characteristics and Outcomes of 1591 Patients Infected With SARS-CoV-2 Admitted to ICUs of the Lombardy Region, Italy. JAMA. 2020;323:1574-81. 10.1001/jama.2020.5394 32250385 PMC7136855

[2] Johns Hopkins University. Johns Hopkins experts in global public health, infectious disease, and emergency preparedness have been at the forefront of the international response to COVID-19. 2021. Available: https://coronavirus.jhu.edu/. Accessed: 11 April 2021.

[3] Rosenberg ES Dufort EM Udo T Wilberschied LA Kumar J Tesoriero J Association of Treatment With Hydroxychloroquine or Azithromycin With In-Hospital Mortality in Patients With COVID-19 in New York State. JAMA. 2020;323:2493-502. 10.1001/jama.2020.8630 32392282 PMC7215635

[4] Manjaly Thomas ZR Leuppi-Taegtmeyer A Jamiolkowski D Steveling-Klein E Bellutti-Enders F Scherer Hofmeier K Emerging treatments in COVID-19: Adverse drug reactions including drug hypersensitivities. J Allergy Clin Immunol. 2020;146:786-9. 10.1016/j.jaci.2020.07.008 32710973 PMC7833501

[5] Feng Y Ling Y Bai T Xie Y Huang J Li J COVID-19 with Different Severities: A Multicenter Study of Clinical Features. Am J Respir Crit Care Med. 2020;201:1380-8. 10.1164/rccm.202002-0445OC 32275452 PMC7258639

[6] Du Y Tu L Zhu P Mu M Wang R Yang P Clinical Features of 85 Fatal Cases of COVID-19 from Wuhan. A Retrospective Observational Study. Am J Respir Crit Care Med. 2020;201:1372-9. 10.1164/rccm.202003-0543OC 32242738 PMC7258652

[7] Scarpignato C Gatta L Zullo A Blandizzi C Effective and safe proton pump inhibitor therapy in acid-related diseases - A position paper addressing benefits and potential harms of acid suppression. BMC Med. 2016;14:179. 10.1186/s12916-016-0718-z 27825371 PMC5101793

[8] Schumock GT Li EC Suda KJ Wiest MD Stubbings J Matusiak LM National trends in prescription drug expenditures and projections for 2016. Am J Health Syst Pharm. 2016;73:1058-75. 10.2146/ajhp160205 27170624

[9] Lilly CM Aljawadi M Badawi O Onukwugha E Tom SE Magder LS Comparative Effectiveness of Proton Pump Inhibitors vs Histamine Type 2 Receptor Blockers for Preventing Clinically Important Gastrointestinal Bleeding During Intensive Care: A Population-Based Study. Chest. 2018;154:557-66. 10.1016/j.chest.2018.05.015 29856970 PMC6689080

[10] MacLaren R Reynolds PM Allen RR Histamine-2 receptor antagonists vs proton pump inhibitors on gastrointestinal tract hemorrhage and infectious complications in the intensive care unit. JAMA Intern Med. 2014;174:564-74. 10.1001/jamainternmed.2013.14673 24535015

[11] Kurlander JE Rubenstein J Richardson C Krein S De Vries R Zikmund-Fisher B Physicians’ Perceptions of Proton Pump Inhibitor Risks and Recommendations to Discontinue: A National Survey. Am J Gastroenterol. 2020;115:689-96. 10.14309/ajg.0000000000000558 32091419 PMC7196016

[12] Wu Z McGoogan JM Characteristics of and Important Lessons From the Coronavirus Disease 2019 (COVID-19) Outbreak in China: Summary of a Report of 72 314 Cases From the Chinese Center for Disease Control and Prevention. JAMA. 2020;323:1239-42. 10.1001/jama.2020.2648 32091533

[13] Grasselli G Pesenti A Cecconi M Critical Care Utilization for the COVID-19 Outbreak in Lombardy, Italy: Early Experience and Forecast During an Emergency Response. JAMA. 2020;323:1545-6. 10.1001/jama.2020.4031 32167538

[14] Yang X Yu Y Xu J Shu H Xia J Liu H Clinical course and outcomes of critically ill patients with SARS-CoV-2 pneumonia in Wuhan, China: a single-centered, retrospective, observational study. Lancet Respir Med. 2020;8:475-81. 10.1016/S2213-2600(20)30079-5 32105632 PMC7102538

[15] Haudebourg AF Perier F Tuffet S de Prost N Razazi K Mekontso Dessap A Respiratory Mechanics of COVID-19- versus Non-COVID-19-associated Acute Respiratory Distress Syndrome. Am J Respir Crit Care Med. 2020;202:287-90. 10.1164/rccm.202004-1226LE 32479162 PMC7365370

[16] Goel K Gupta T Kolte D Khera S Fonarow GC Bhatt DL Outcomes and Temporal Trends of Inpatient Percutaneous Coronary Intervention at Centers With and Without On-site Cardiac Surgery in the United States. JAMA Cardiol. 2017;2:25-33. 10.1001/jamacardio.2016.4188 27893054

[17] Gupta T Kolte D Khera S Goel K Aronow WS Cooper HA Management and Outcomes of ST-Segment Elevation Myocardial Infarction in US Renal Transplant Recipients. JAMA Cardiol. 2017;2:250-8. 10.1001/jamacardio.2016.5131 28097322

[18] Woo A Lee SW Koh HY Kim MA Han MY Yon DK Incidence of cancer after asthma development: 2 independent population-based cohort studies. J Allergy Clin Immunol. 2021;147:135-43. 10.1016/j.jaci.2020.04.041 32417133

[19] Arendse LB Danser AHJ Poglitsch M Touyz RM Burnett JC Jr Llorens-Cortes C Novel Therapeutic Approaches Targeting the Renin-Angiotensin System and Associated Peptides in Hypertension and Heart Failure. Pharmacol Rev. 2019;71:539-70. 10.1124/pr.118.017129 31537750 PMC6782023

[20] Effenberger M Grabherr F Mayr L Schwaerzler J Nairz M Seifert M Faecal calprotectin indicates intestinal inflammation in COVID-19. Gut. 2020;69:1543-4. 10.1136/gutjnl-2020-321388 32312790 PMC7211078

[21] Lipworth B Kuo CR Lipworth S Chan R Inhaled Corticosteroids and COVID-19. Am J Respir Crit Care Med. 2020;202:899-900. 10.1164/rccm.202005-2000LE 32668178 PMC7491401

[22] Zhang H Rostami MR Leopold PL Mezey JG O’Beirne SL Strulovici-Barel Y Expression of the SARS-CoV-2 ACE2 Receptor in the Human Airway Epithelium. Am J Respir Crit Care Med. 2020;202:219-29. 10.1164/rccm.202003-0541OC 32432483 PMC7365377

[23] Lin L Jiang X Zhang Z Huang S Zhang Z Fang Z Gastrointestinal symptoms of 95 cases with SARS-CoV-2 infection. Gut. 2020;69:997-1001. 10.1136/gutjnl-2020-321013 32241899

[24] Hoffmann M Kleine-Weber H Schroeder S Krüger N Herrler T Erichsen S SARS-CoV-2 Cell Entry Depends on ACE2 and TMPRSS2 and Is Blocked by a Clinically Proven Protease Inhibitor. Cell. 2020;181:271-80.e8. 10.1016/j.cell.2020.02.052 32142651 PMC7102627

[25] Lebreton G Dorgham K Quentric P Combes A Gorochov G Schmidt M Longitudinal Cytokine Profiling in Severe COVID-19 Patients on ECMO and Haemoadsorption. Am J Respir Crit Care Med. 2021;203:1433-5. 10.1164/rccm.202011-4140LE 33725469 PMC8456531

[26] Saheb Sharif-Askari N Saheb Sharif-Askari F Alabed M Tayoun AA Loney T Uddin M Effect of Common Medications on the Expression of SARS-CoV-2 Entry Receptors in Kidney Tissue. Clin Transl Sci. 2020;13:1048-54. 10.1111/cts.12862 32799423 PMC7461457

[27] Lee SW Ha EK Yeniova A Moon SY Kim SY Koh HY Severe clinical outcomes of COVID-19 associated with proton pump inhibitors: a nationwide cohort study with propensity score matching. Gut. 2021;70:76-84. 10.1136/gutjnl-2020-322248 32732368

[28] Monteleone G Sarzi-Puttini PC Ardizzone S Preventing COVID-19-induced pneumonia with anticytokine therapy. Lancet Rheumatol. 2020;2:e255-6. 10.1016/S2665-9913(20)30092-8 32368737 PMC7193140

[29] Zhou J Li C Zhao G Chu H Wang D Yan HH Human intestinal tract serves as an alternative infection route for Middle East respiratory syndrome coronavirus. Sci Adv. 2017;3:eaao4966. 10.1126/sciadv.aao4966 29152574 PMC5687858

[30] Luxenburger H Sturm L Biever P Rieg S Duerschmied D Schultheiss M Treatment with proton pump inhibitors increases the risk of secondary infections and ARDS in hospitalized patients with COVID-19: coincidence or underestimated risk factor? J Intern Med. 2021;289:121-4. 10.1111/joim.13121 32608546 PMC7361636

[31] Hariyanto TI Prasetya IB Kurniawan A Proton pump inhibitor use is associated with increased risk of severity and mortality from coronavirus disease 2019 (COVID-19) infection. Dig Liver Dis. 2020;52:1410-2. 10.1016/j.dld.2020.10.001 33092998 PMC7538064

[32] Guan WJ Liang WH Zhao Y Liang HR Chen ZS Li YM Comorbidity and its impact on 1590 patients with COVID-19 in China: a nationwide analysis. Eur Respir J. 2020;55:2000547. 10.1183/13993003.00547-2020 32217650 PMC7098485

[33] Lighter J Phillips M Hochman S Sterling S Johnson D Francois F Obesity in Patients Younger Than 60 Years Is a Risk Factor for COVID-19 Hospital Admission. Clin Infect Dis. 2020;71:896-7. 10.1093/cid/ciaa415 32271368 PMC7184372

[34] Dai M Liu D Liu M Zhou F Li G Chen Z Patients with Cancer Appear More Vulnerable to SARS-CoV-2: A Multicenter Study during the COVID-19 Outbreak. Cancer Discov. 2020;10:783-91. 32345594 10.1158/2159-8290.CD-20-0422 PMC7309152

[35] Blanco JL Ambrosioni J Garcia F Martínez E Soriano A Mallolas J COVID-19 in patients with HIV: clinical case series. Lancet HIV. 2020;7:e314-6. 10.1016/S2352-3018(20)30111-9 32304642 PMC7159872

