# Supplementary material for: Use of proton pump inhibitors are associated with higher mortality in hospitalized patients with COVID-19
Source: J Glob Health. 2022 Feb 19;12:05005. doi: 10.7189/jogh.12.05005 (PMC8849264; doi:10.7189/jogh.12.05005)
Supplement: Online Supplementary Document [file jogh-12-05005-s001.pdf]

Table S1 Missing information of all patients who confirmed COVID-19 in cohort

Table S1 Missing information of all patients who confirmed COVID-19 in cohort

Characteristic	Missing number	Missing percentage (%)	Value of imputation
Age group, years	0	0.00%	-
Sex	0	0.00%	-
Medical insurance	488	10.53%	Medical insurance for urban
Severity of illness	1	0.02%	Minor
Admission temperature	0	0.00%	-
Systolic blood pressure	10	0.22%	Normal
Diastolic blood pressure	17	0.37%	Normal
Pulse rate	3	0.06%	60-100 p.m.
White blood cell count	51	1.10%	⩽9.5×10^9/L
Red blood cell count*	51	1.10%	Normal
Mononuclear leucocyte count	52	1.12%	＞0.6×10^9/L
Neutrophil count	52	1.12%	⩽7×10^9/L
Lymphocyte count	51	1.10%	⩽4×10^9/L
Thrombocyte count	55	1.19%	⩾100×10^9/L
Glutamic-pyruvic transaminase**	144	3.11%	Normal
Glutamic-oxalacetic transaminase	135	2.91%	Normal
Serum creatinine	195	4.21%	⩽73 μmol/L
Blood urea nitrogen	195	4.21%	⩽8 μmol/L
Serum albumin	139	3.00%	Normal
Serum globulin	685	14.78%	Normal
Total bilirubin	139	3.00%	＞19μmol/L
Alkaline phosphatase	140	3.02%	＞100 IU/L
Oxyhemoglobin saturation	182	3.93%	⩾90%
History of hypertension	0	0.00%	-
History of diabetes mellitus	17	0.37%	No
History of malignant tumor	0	0.00%	-
History of stroke	0	0.00%	-
History of COPD	0	0.00%	-
History of coronary heart disease	0	0.00%	-
History of digestive system disease	0	0.00%	-
Antiviral treatment	0	0.00%	-

Table S1 Missing information of all patients who confirmed COVID-19 in cohort

Characteristic	Missing number	Missing percentage (%)	Value of imputation
Antibiotic therapy	0	0.00%	-
Antifungal therapy	0	0.00%	-
Immunotherapy	0	0.00%	-
Hormonotherapy	0	0.00%	-
Plasma therapy	0	0.00%	-
Transfer to ICU	0	0.00%	-
Death	0	0.00%	-
LOS	0	0.00%	-
The time of COVID-19 nucleic acid testing turning negative	80	1.73%	38 days

* Male: normal 4.0-5.5×10^^{12}/L, higher >5.5×10^^{12}/L, lower <4.0×10^^{12}/L

Female: normal 3.5-5.0×10^^{12}/L, higher >5.0×10^^{12}/L, lower <3.5×10^^{12}/L

**Normal 7-40 IU/L, abnormal <7 IU/L or >40 IU/L

*** Normal 7-45 IU/L, abnormal <7 IU/L or >45 IU/L

Table S2 Baseline characteristics of all patients who confirmed COVID-19 in cohort (N=4 634)

Table S2 Baseline characteristics of all patients who confirmed COVID-19 in cohort (N=4 634).

Characteristic	Entire cohort	Without use of PPIs	With use of PPIs	P value
Total, n (%)	4634(100.00)	3588(100.00)	1046(100.00)	
Age group, years, n (%)				<.0001
18-40	609(13.14)	556(15.50)	53(5.07)	
41-65	2478(53.47)	1963(54.71)	515(49.24)	
⩾65	1547(33.38)	1069(29.79)	478(45.70)	
Sex, n (%)				<.0001
Female	2412(52.05)	1780(49.61)	632(60.42)	
Male	2222(47.95)	1808(50.39)	414(39.58)	
Medical insurance, n (%)				0.4190
Medical insurance for urban	2312(49.89)	1788(49.83)	524(50.10)	
New rural cooperative medical insurance	69(1.49)	50(1.39)	19(1.82)	
Free medical treatment	598(12.90)	475(13.24)	123(11.76)	
Self-pay	372(8.03)	295(8.22)	77(7.36)	
Other	1283(27.69)	980(27.31)	303(28.97)	
Severity of illness, n (%)				<.0001

Table S2 Baseline characteristics of all patients who confirmed COVID-19 in cohort (N=4 634).

Characteristic	Entire cohort	Without use of PPIs	With use of PPIs	P value
Minor	3715(80.17)	3020(84.17)	695(66.44)	
Serious	919(19.83)	568(15.83)	351(33.56)	
Admission temperature, n (%)				0.0028
≤37.0℃	4346(93.79)	3386(94.37)	960(91.78)	
＞37.0℃	288(6.21)	202(5.63)	86(8.22)	
Systolic blood pressure, n (%)*				0.3264
Normal	3504(75.62)	2725(75.95)	779(74.47)	
Abnormal	1130(24.38)	863(24.05)	267(25.53)	
Diastolic blood pressure, n (%)*				0.0098
Normal	3696(79.76)	2832(78.93)	864(82.60)	
Abnormal	938(20.24)	756(21.07)	182(17.40)	
Pulse rate, n (%)*				0.7625
Normal	3978(85.84)	3083(85.93)	895(85.56)	
Abnormal	656(14.16)	505(14.07)	151(14.44)	
White blood cell count, n (%)				<.0001
≤$9.5\times10^{\wedge 9}$/L	4357(94.02)	3433(95.68)	924(88.34)	
＞$9.5\times10^{\wedge 9}$/L	277(5.98)	155(4.32)	122(11.66)	
Red blood cell count, n (%)#				0.0062

Table S2 Baseline characteristics of all patients who confirmed COVID-19 in cohort (N=4 634).

Characteristic	Entire cohort	Without use of PPIs	With use of PPIs	P value
Normal	2705(58.37)	2129(59.34)	576(55.07)	
Lower	1886(40.70)	1421(39.60)	465(44.46)	
Higher	43(0.93)	38(1.06)	5(0.48)	
Mononuclear leucocyte count, n (%)				0.2134
＞0.6×10^9/L	4497(97.04)	3488(97.21)	1009(96.46)	
⩽0.6×10^9/L	137(2.96)	100(2.79)	37(3.54)	
Neutrophil count, n (%)				<.0001
⩽7×10^9/L	4343(93.72)	3440(95.88)	903(86.33)	
＞7×10^9/L	291(6.28)	148(4.12)	143(13.67)	
Lymphocyte count, n (%)				0.5609
⩽4×10^9/L	4566(98.53)	3533(98.47)	1033(98.76)	
＞4×10^9/L	68(1.47)	55(1.53)	13(1.24)	
Thrombocyte count, n (%)				<.0001
⩾100×10^9/L	4511(97.35)	3520(98.10)	991(94.74)	
＜100×10^9/L	123(2.65)	68(1.90)	55(5.26)	
Glutamic-pyruvic transaminase, n (%)*				0.5264
Normal	3604(77.77)	2798(77.98)	806(77.06)	
Abnormal	1030(22.23)	790(22.02)	240(22.94)	

Table S2 Baseline characteristics of all patients who confirmed COVID-19 in cohort (N=4 634).

Characteristic	Entire cohort	Without use of PPIs	With use of PPIs	P value
Glutamic-oxalacetic transaminase, n (%) *				<.0001
Normal	4331(93.46)	3391(94.51)	940(89.87)	
Abnormal	303(6.54)	197(5.49)	106(10.13)	
Serum creatinine, n (%)				0.2799
≤73 μmol/L	3088(66.64)	2376(66.22)	712(68.07)	
＞73 μmol/L	1546(33.36)	1212(33.78)	334(31.93)	
Blood urea nitrogen, n (%)				<.0001
≤8 μmol/L	4306(92.92)	3410(95.04)	896(85.66)	
＞8 μmol/L	328(7.08)	178(4.96)	150(14.34)	
Serum albumin, n (%)*				<.0001
Normal	3610(77.90)	2949(82.19)	661(63.19)	
Abnormal	1024(22.09)	639(17.81)	385(36.81)	
Serum globulin, n (%)*				0.00027
Normal	2982(64.35)	2361(65.80)	621(59.37)	
Abnormal	1652(35.65)	1227(34.20)	425(40.63)	
Total bilirubin, n (%)				0.0644
＞19μmol/L	4355(93.98)	3385(94.34)	970(92.73)	
≤19μmol/L	279(6.02)	203(5.66)	76(7.27)	

Table S2 Baseline characteristics of all patients who confirmed COVID-19 in cohort (N=4 634).

Characteristic	Entire cohort	Without use of PPIs	With use of PPIs	P value
Alkaline phosphatase, n (%)				0.0002
＞100 IU/L	4107(88.63)	3215(89.60)	892(85.28)	
⩽100 IU/L	527(11.37)	373(10.40)	154(14.72)	
Oxyhemoglobin saturation, n (%)				<.0001
⩾90%	4581(98.86)	3563(99.30)	1018(97.32)	
＜90%	53(1.14)	25(0.70)	28(2.68)	
History of hypertension, n (%)	1477(31.87)	1091(30.41)	386(36.90)	<.0001
History of diabetes mellitus, n (%)	628(13.55)	451(12.57)	177(16.92)	0.0004
History of malignant tumor, n (%)	42(0.91)	30(0.84)	12(1.15)	0.3549
History of stroke, n (%)	148(3.19)	95(2.65)	53(5.07)	0.0002
History of COPD, n (%)	48(1.04)	25(0.70)	23(2.20)	0.0002
History of coronary heart disease, n (%)	281(6.06)	165(4.60)	116(11.09)	<.0001
History of digestive system disease, n (%)	459(9.91)	278(7.75)	181(17.30)	<.0001
Antiviral treatment, n (%)	2733(58.98)	2032(56.63)	701(67.02)	<.0001
Antibiotic therapy, n (%)	1614(34.83)	1056(29.43)	558(53.35)	<.0001
Antifungal therapy, n (%)	40(0.86)	6(0.17)	34(3.25)	<.0001
Immunotherapy, n (%)	749(16.16)	499(13.91)	250(23.90)	<.0001

Table S2 Baseline characteristics of all patients who confirmed COVID-19 in cohort (N=4 634).

Characteristic	Entire cohort	Without use of PPIs	With use of PPIs	P value
Hormonotherapy, n (%)	546(11.78)	231(6.44)	315(30.11)	<.0001
Transfer to ICU, n (%)	67(1.45)	17(0.47)	50(4.78)	<.0001
Death, n (%)	92(1.99)	19(0.53)	73(6.98)	<.0001

* Systolic blood pressure

Normal: 90-140 mmHg, abnormal: <90 mmHg or >140 mmHg

* Diastolic blood pressure

Normal: 60-90 mmHg, abnormal: <60 mmHg or >90 mmHg

* Pulse rate

Normal: 60-100 p.m., abnormal: ＜60 p.m. or ＞100 p.m.

* Glutamic-pyruvic transaminase

Normal 7-40 IU/L, abnormal <7 IU/L or >40 IU/L

* Glutamic-oxalacetic transaminase

Normal 7-45 IU/L, abnormal <7 IU/L or >45 IU/L

* Serum albumin

Normal: 35-55 g/L, abnormal: ＜35 g/L or >55 IU/L

* Serum globulin

Normal: 20-30 g/L, abnormal: ＜20 g/L or >30 IU/L

Male: normal 4.0-5.5×10^^{12}/L, higher >5.5×10^^{12}/L, lower <4.0×10^^{12}/L

Female: normal 3.5-5.0×10^^{12}/L, higher >5.0×10^^{12}/L, lower <3.5×10^^{12}/L

Table S3 Outcomes of the univariate logistic regression and 4 models

Table S3 Outcomes of the univariate logistic regression and 4 models.

Characteristic	Univariate Logistic Regression		Model 1		Model 2	
	OR (95%CI)	P value	OR (95%CI)	P value	OR (95%CI)	P value
PPIs usage	14.09(8.47-23.46)	<0.0001	3.63(1.83-7.23)	0.0002	-	-
PPIs dose						
Non	Ref				Ref	
⩽ 0.5 DDD	6.33(3.10-12.91)	<0.0001			2.97(1.18-7.51)	0.0214
1 DDD	17.42(10.02-30.29)	<0.0001			3.59(1.70-7.59)	0.0008
⩾ 1.5DDD	24.61(12.76-47.49)	<0.0001			4.95(1.96-12.47)	0.0007
Age group, years						

Table S3 Outcomes of the univariate logistic regression and 4 models.

Characteristic	Univariate Logistic Regression		Model 1		Model 2	
	OR (95%CI)	P value	OR (95%CI)	P value	OR (95%CI)	P value
18-40	Ref		Ref		Ref	
41-65	4.95(0.66-36.91)	0.1191	1.80(0.22-14.99)	0.5850	1.78(0.21-14.81)	0.5927
⩾65	29.24(4.05-210.84)	0.0008	4.21(0.53-33.51)	0.1743	4.20(0.53-33.43)	0.1748
Sex						
Female	Ref					
Male	2.17(1.40-3.35)	0.0005				
Medical insurance						
Medical insurance for urban	Ref					

Table S3 Outcomes of the univariate logistic regression and 4 models.

Characteristic	Univariate Logistic Regression		Model 1		Model 2	
	OR (95%CI)	P value	OR (95%CI)	P value	OR (95%CI)	P value
New rural cooperative medical insurance	0.71(0.10-5.21)	0.7352				
Free medical treatment	0.57(0.26-1.27)	0.1691				
Self-pay	0.92(0.41-2.06)	0.8472				
Other	1.15(0.73-1.83)	0.5449				
Severity of illness						
Minor	Ref		Ref		Ref	
Serious	15.70(9.52-25.92)	<0.0001	1.89(0.93-3.81)	0.0774	1.84(0.91-3.74)	0.0899
Admission temperature						

Table S3 Outcomes of the univariate logistic regression and 4 models.

Characteristic	Univariate Logistic Regression		Model 1		Model 2	
	OR (95%CI)	P value	OR (95%CI)	P value	OR (95%CI)	P value
≤37.0℃	Ref		Ref		Ref	
＞37.0℃	4.43(2.66-7.38)	<0.0001	2.31(1.10-4.85)	0.0263	2.27(1.08-4.77)	0.0296
Systolic blood pressure						
Normal	Ref					
Abnormal	1.59(1.03-2.47)	0.0373				
Diastolic blood pressure						
Normal	Ref					
Abnormal	1.03(0.62-1.71)	0.9206				
Pulse rate						

Table S3 Outcomes of the univariate logistic regression and 4 models.

Characteristic	Univariate Logistic Regression		Model 1		Model 2	
	OR (95%CI)	P value	OR (95%CI)	P value	OR (95%CI)	P value
Normal	Ref					
Abnormal	0.78(0.45-1.34)	0.3693				
White blood cell count						
⩽9.5×10^9/L	Ref					
＞9.5×10^9/L	10.37(6.66-16.15)	<0.0001				
Red blood cell count*						
Normal	Ref					
Lower	3.14(2.01-4.89)	<0.0001				
Higher	2.20(0.29-16.51)	0.4443				

Table S3 Outcomes of the univariate logistic regression and 4 models.

Characteristic	Univariate Logistic Regression		Model 1		Model 2	
	OR (95%CI)	P value	OR (95%CI)	P value	OR (95%CI)	P value
Mononuclear leucocyte count						
＞0.6×10^9/L	Ref					
⩽0.6×10^9/L	2.80(1.27-6.16)	0.0108				
Neutrophil count						
⩽7×10^9/L	Ref		Ref		Ref	
＞7×10^9/L	15.94(10.38-24.47)	<0.0001	2.11(1.15-3.86)	0.0155	2.11(1.15-3.86)	0.0156
Lymphocyte count						
⩽4×10^9/L	Ref		Ref		Ref	
＞4×10^9/L	5.04(2.12-11.97)	0.0002	44.70(12.63-158.21)	<0.0001	45.68(12.94-161.30)	<0.0001

Table S3 Outcomes of the univariate logistic regression and 4 models.

Characteristic	Univariate Logistic Regression		Model 1		Model 2	
	OR (95%CI)	P value	OR (95%CI)	P value	OR (95%CI)	P value
Thrombocyte count						
⩾100×10^9/L	Ref		Ref		Ref	
＜100×10^9/L	20.48(12.57-33.38)	<0.0001	5.63(2.73-11.58)	<0.0001	5.65(2.73-11.69)	<0.0001
Glutamic-pyruvic transaminase**						
Normal	Ref					
Abnormal	1.63(1.04-2.54)	0.0319				
Glutamic-oxalacetic transaminase						

Table S3 Outcomes of the univariate logistic regression and 4 models.

Characteristic	Univariate Logistic Regression		Model 1		Model 2	
	OR (95%CI)	P value	OR (95%CI)	P value	OR (95%CI)	P value
Normal	Ref					
Abnormal	6.07(3.79-9.70)	<0.0001				
Serum creatinine						
≤73 μmol/L	Ref					
＞73 μmol/L	2.91(1.91-4.42)	<0.0001				
Blood urea nitrogen						
≤8 μmol/L	Ref		Ref		Ref	
＞8 μmol/L	15.84(10.34-24.25)	<0.0001	2.44(1.33-4.46)	0.0038	2.36(1.28-4.33)	0.0057
Serum albumin						

Table S3 Outcomes of the univariate logistic regression and 4 models.

Characteristic	Univariate Logistic Regression		Model 1		Model 2	
	OR (95%CI)	P value	OR (95%CI)	P value	OR (95%CI)	P value
Normal	Ref					
Abnormal	10.04(6.313-15.98)	<0.0001				
Serum globulin						
Normal	Ref					
Abnormal	0.96(0.62-1.48)	0.8615				
Total bilirubin						
＞19μmol/L	Ref		Ref		Ref	
⩽19μmol/L	4.91(2.97-8.12)	<0.0001	2.13(1.03-4.39)	0.0405	2.16(1.05-4.46)	0.0369
Alkaline phosphatase						

Table S3 Outcomes of the univariate logistic regression and 4 models.

Characteristic	Univariate Logistic Regression		Model 1		Model 2	
	OR (95%CI)	P value	OR (95%CI)	P value	OR (95%CI)	P value
＞100 IU/L	Ref					
⩽100 IU/L	4.15(2.66-6.45)	<0.0001				
Oxyhemoglobin saturation						
⩾90%	Ref		Ref		Ref	
＜90%	34.51(18.80-63.33)	<0.0001	6.58(2.77-15.66)	<0.0001	6.60(2.77-15.77)	<0.0001
History of hypertension	2.27(1.50-3.44)	<0.0001				
History of diabetes mellitus	2.17(1.34-3.50)	0.0015				
History of malignant tumor	3.89(1.18-12.83)	0.0256				
History of stroke	5.37(2.92-9.90)	<0.0001	2.92(1.25-6.84)	0.0137	2.92(1.25-6.86)	0.0137

Table S3 Outcomes of the univariate logistic regression and 4 models.

Characteristic	Univariate Logistic Regression		Model 1		Model 2	
	OR (95%CI)	P value	OR (95%CI)	P value	OR (95%CI)	P value
History of COPD	6.01(2.33-15.55)	0.0002				
History of coronary heart disease	4.87(2.95-8.05)	<0.0001				
History of digestive system disease	2.10(1.23-3.59)	0.0065				
Antiviral treatment	1.78(1.13-2.82)	0.0131				
Antibiotic therapy	34.35(13.92-84.76)	<0.0001	5.65(2.04-15.67)	0.0009	5.50(1.97-15.32)	0.0011
Antifungal therapy	44.54(22.85-86.78)	<0.0001				
Immunotherapy	3.45(2.25-5.29)	<0.0001				

Table S3 Outcomes of the univariate logistic regression and 4 models.

Characteristic	Univariate Logistic Regression		Model 1		Model 2	
	OR (95%CI)	P value	OR (95%CI)	P value	OR (95%CI)	P value
Hormonotherapy	27.18(16.68-44.29)	<0.0001	3.27(1.67-6.39)	0.0005	3.24(1.65-6.34)	0.0006
Transfer to ICU	74.16(43.07-127.70)	<0.0001	7.64(3.79-15.40)	<0.0001	7.80(3.86-15.76)	<0.0001

Table S3 (Continued) Outcomes of the univariate logistic regression and 4 models.

Characteristic	Model 3		Model 4	
	OR (95%CI)	P value	OR (95%CI)	P value
PPIs usage	2.95(1.57-5.52)	0.0008	2.97(1.63-5.42)	0.0004
PPIs dose				
Non				
Low				
High				
Age group, years				
18-40				
41-65				
⩾65				

Table S3 (Continued) Outcomes of the univariate logistic regression and 4 models.

Characteristic	Model 3		Model 4	
	OR (95%CI)	P value	OR (95%CI)	P value
Sex				
Female				
Male				
Medical insurance				
Medical insurance for urban				
New rural cooperative medical insurance				
Free medical treatment				
Self-pay				

Table S3 (Continued) Outcomes of the univariate logistic regression and 4 models.

Characteristic	Model 3		Model 4	
	OR (95%CI)	P value	OR (95%CI)	P value
Other				
Severity of illness				
Minor				
Serious			2.18(1.11-4.28)	0.0242
Admission temperature				
≤37.0℃				
＞37.0℃			2.99(1.55-5.76)	0.0011
Systolic blood pressure				
Normal				

Table S3 (Continued) Outcomes of the univariate logistic regression and 4 models.

Characteristic	Model 3		Model 4	
	OR (95%CI)	P value	OR (95%CI)	P value
Abnormal			2.06(1.14-3.73)	0.0173
Diastolic blood pressure				
Normal				
Abnormal				
Pulse rate				
Normal				
Abnormal				
White blood cell count				
⩽9.5×10^9/L				

Table S3 (Continued) Outcomes of the univariate logistic regression and 4 models.

Characteristic	Model 3		Model 4	
	OR (95%CI)	P value	OR (95%CI)	P value
＞9.5×10^9/L				
Red blood cell count*				
Normal				
Lower				
Higher				
Mononuclear leucocyte count				
＞0.6×10^9/L				
⩽0.6×10^9/L				
Neutrophil count				

Table S3 (Continued) Outcomes of the univariate logistic regression and 4 models.

Characteristic	Model 3		Model 4	
	OR (95%CI)	P value	OR (95%CI)	P value
≤7×10^{9}/L				
>7×10^{9}/L			2.18(1.27-3.75)	0.0049
Lymphocyte count				
≤4×10^{9}/L				
>4×10^{9}/L			18.73(3.01-116.52)	0.0017
Thrombocyte count				
≥100×10^{9}/L				
<100×10^{9}/L			5.37(2.74-10.55)	<0.0001

Table S3 (Continued) Outcomes of the univariate logistic regression and 4 models.

Characteristic	Model 3		Model 4	
	OR (95%CI)	P value	OR (95%CI)	P value
Glutamic-pyruvic transaminase**				
Normal				
Abnormal				
Glutamic-oxalacetic transaminase				
Normal				
Abnormal				
Serum creatinine				

Table S3 (Continued) Outcomes of the univariate logistic regression and 4 models.

Characteristic	Model 3		Model 4	
	OR (95%CI)	P value	OR (95%CI)	P value
≤73 μmol/L				
＞73 μmol/L			3.22(1.84-5.64)	<0.0001
Blood urea nitrogen				
≤8 μmol/L				
＞8 μmol/L				
Serum albumin				
Normal				
Abnormal				
Serum globulin				

Table S3 (Continued) Outcomes of the univariate logistic regression and 4 models.

Characteristic	Model 3		Model 4	
	OR (95%CI)	P value	OR (95%CI)	P value
Normal				
Abnormal				
Total bilirubin				
＞19μmol/L				
⩽19μmol/L			2.32(1.15-4.69)	0.0185
Alkaline phosphatase				
＞100 IU/L				
⩽100 IU/L				
Oxyhemoglobin saturation				

Table S3 (Continued) Outcomes of the univariate logistic regression and 4 models.

Characteristic	Model 3		Model 4	
	OR (95%CI)	P value	OR (95%CI)	P value
⩾90%				
＜90%			6.08(2.81-13.16)	<0.0001
History of hypertension			0.37(0.20-0.66)	0.0009
History of diabetes mellitus				
History of malignant tumor				
History of stroke			5.48(2.57-11.68)	<0.0001
History of COPD				
History of coronary heart disease			4.31(2.23-8.33)	<0.0001

Table S3 (Continued) Outcomes of the univariate logistic regression and 4 models.

Characteristic	Model 3		Model 4	
	OR (95%CI)	P value	OR (95%CI)	P value
History of digestive system disease				
Antiviral treatment				
Antibiotic therapy			17.36(3.29-91.66)	0.0008
Antifungal therapy			2.60(1.11-6.10)	0.0277
Immunotherapy				
Hormonotherapy			3.30(1.72-6.36)	0.0003
Transfer to ICU			7.00(3.69-13.27)	<0.0001

Table S4 Baseline characteristics and SMD of patients after propensity score matching and propensity score weighting

Table S4 Baseline characteristics and SMD of patients after propensity score matching and propensity score weighting.

Characteristic	Primary data			After propensity score matching			After propensity score weighting		
	With use of PPIs	Without use of PPIs	SMD	With use of PPIs	Without use of PPIs	SMD	With use of PPIs	Without use of PPIs	SMD
Total, n (%)	3588(77.43)	1046(22.57)	-	1560(63.99)	878(36.01)	-	1052(50.14)	1046(49.86)	-
Age group, years, n (%)			-			-			-
18-40	556(15.50)	53(5.07)	0.10	103(6.60)	52(5.92)	<0.01	54(5.13)	53(5.07)	<0.01
41-65	1963(54.71)	515(49.24)	0.05	836(53.59)	465(52.96)	<0.01	507(48.15)	515(49.24)	0.01
⩾65	1069(29.79)	478(45.70)	0.16	621(39.81)	361(41.12)	<0.01	492(46.72)	478(45.70)	0.01
Sex, n (%)			0.11			<0.01			<0.01
Female	1780(49.61)	632(60.42)	-	957(61.35)	540(61.50)	-	635(60.32)	632(60.42)	-
Male	1808(50.39)	414(39.58)	-	603(38.65)	338(38.50)	-	418(39.68)	414(39.58)	-
Medical insurance, n (%)			-			-			-
Medical insurance for urban	1788(49.83)	524(50.10)	<0.01	750(48.08)	435(49.54)	0.01	503(47.82)	524(50.10)	0.02
New rural cooperative medical insurance	50(1.39)	19(1.82)	<0.01	28(1.79)	15(1.71)	<0.01	15(1.44)	19(1.82)	<0.01
Free medical treatment	475(13.24)	123(11.76)	0.01	215(13.78)	106(12.07)	0.01	125(11.84)	123(11.76)	<0.01
Self-pay	295(8.22)	77(7.36)	<0.01	123(7.88)	71(8.09)	<0.01	82(7.81)	77(7.36)	<0.01
Other	980(27.31)	303(28.97)	0.02	444(28.46)	251(28.59)	<0.01	327(31.09)	303(28.97)	0.02

Table S4 Baseline characteristics and SMD of patients after propensity score matching and propensity score weighting.

Characteristic	Primary data			After propensity score matching			After propensity score weighting		
	With use of PPIs	Without use of PPIs	SMD	With use of PPIs	Without use of PPIs	SMD	With use of PPIs	Without use of PPIs	SMD
Severity of illness, n (%)			0.18			0.01			0.01
Minor	3020(84.17)	695(66.44)	-	1222(78.33)	650(74.03)	-	712(67.68)	695(66.44)	-
Serious	568(15.83)	351(33.56)	-	338(21.67)	228(25.97)	-	340(32.32)	351(33.56)	-
Admission temperature, n (%)			0.03			<0.01			<0.01
≤37.0℃	3386(94.37)	960(91.78)	-	1460(93.59)	824(93.85)	-	976(92.72)	960(91.78)	-
＞37.0℃	202(5.63)	86(8.22)	-	100(6.41)	54(6.15)	-	77(7.28)	86(8.22)	-
Systolic blood pressure, n (%)*			0.01			<0.01			0.01
Normal	2725(75.95)	779(74.47)	-	1162(74.49)	658(74.94)	-	798(75.83)	779(74.47)	-
Abnormal	863(24.05)	267(25.53)	-	398(25.51)	220(25.06)	-	254(24.17)	267(25.53)	-
Diastolic blood pressure, n (%)*			0.04			<0.01			<0.01
Normal	2832(78.93)	864(82.60)	-	1289(82.63)	724(82.46)	-	869(82.56)	864(82.60)	-
Abnormal	756(21.07)	182(17.40)	-	271(17.37)	154(17.54)	-	184(17.44)	182(17.40)	-
Pulse rate, n (%)*			<0.01			0.01			0.01
Normal	3083(85.93)	895(85.56)	-	1336(85.64)	761(86.67)	-	916(87.06)	895(85.56)	-
Abnormal	505(14.07)	151(14.44)	-	224(14.36)	117(13.33)	-	136(12.94)	151(14.44)	-
White blood cell count, n (%)			0.07			<0.01			<0.01
≤9.5×10^^{9}/L	3433(95.68)	924(88.34)	-	1451(93.01)	803(91.46)	-	940(89.30)	924(88.34)	-
＞9.5×10^^{9}/L	155(4.32)	122(11.66)	-	109(6.99)	75(8.54)	-	113(10.70)	122(11.66)	-

Table S4 Baseline characteristics and SMD of patients after propensity score matching and propensity score weighting.

Characteristic	Primary data			After propensity score matching			After propensity score weighting		
	With use of PPIs	Without use of PPIs	SMD	With use of PPIs	Without use of PPIs	SMD	With use of PPIs	Without use of PPIs	SMD
Red blood cell count, n (%)[#]			-			-			-
Normal	2129(59.34)	576(55.07)	0.04	943(60.45)	513(58.43)	0.01	597(56.75)	576(55.07)	0.02
Lower	1421(39.60)	465(44.46)	0.05	610(39.10)	362(41.23)	0.01	451(42.88)	465(44.46)	0.02
Higher	38(1.06)	5(0.48)	<0.01	7(0.45)	3(0.34)	<0.01	4(0.37)	5(0.48)	<0.01
Mononuclear leucocyte count, n (%)			<0.01			<0.01			<0.01
＞0.6×10^9/L	3488(97.21)	1009(96.46)	-	1516(97.18)	851(96.92)	-	1017(96.65)	1009(96.46)	-
⩽0.6×10^9/L	100(2.79)	37(3.54)	-	44(2.82)	27(3.08)	-	35(3.35)	37(3.54)	-
Neutrophil count, n (%)			0.10			<0.01			<0.01
⩽7×10^9/L	3440(95.88)	903(86.33)	-	1449(92.88)	799(91.00)	-	899(85.42)	903(86.33)	-
＞7×10^9/L	148(4.12)	143(13.67)	-	111(7.12)	79(9.00)	-	153(14.58)	143(13.67)	-
Lymphocyte count, n (%)			<0.01			<0.01			<0.01
⩽4×10^9/L	3533(98.47)	1033(98.76)	-	1541(98.78)	866(98.63)	-	1040(98.82)	1033(98.76)	-
＞4×10^9/L	55(1.53)	13(1.24)	-	19(1.22)	12(1.37)	-	12(1.18)	13(1.24)	-
Thrombocyte count, n (%)			0.03			<0.01			0.01
⩾100×10^9/L	3520(98.10)	991(94.74)	-	1515(97.12)	850(96.81)	-	983(93.37)	991(94.74)	-
＜100×10^9/L	68(1.90)	55(5.26)	-	45(2.88)	28(3.19)	-	70(6.63)	55(5.26)	-
Glutamic-pyruvic transaminase, n (%)*			<0.01			<0.01			0.01
Normal	2798(77.98)	806(77.06)	-	1260(80.77)	696(79.27)	-	826(78.47)	806(77.06)	-

Table S4 Baseline characteristics and SMD of patients after propensity score matching and propensity score weighting.

Characteristic	Primary data			After propensity score matching			After propensity score weighting		
	With use of PPIs	Without use of PPIs	SMD	With use of PPIs	Without use of PPIs	SMD	With use of PPIs	Without use of PPIs	SMD
Abnormal	790(22.02)	240(22.94)	-	300(19.23)	182(20.73)	-	227(21.53)	240(22.94)	-
Glutamic-oxalacetic transaminase, n (%)			0.05			<0.01			<0.01
Normal	3391(94.51)	940(89.87)	-	1454(93.21)	803(91.46)	-	939(89.19)	940(89.87)	-
Abnormal	197(5.49)	106(10.13)	-	106(6.79)	75(8.54)	-	114(10.81)	106(10.13)	-
Serum creatinine, n (%)			0.02			0.01			<0.01
≤73 μmol/L	2376(66.22)	712(68.07)	-	1083(69.42)	617(70.27)	-	718(68.22)	712(68.07)	-
＞73 μmol/L	1212(33.78)	334(31.93)	-	477(30.58)	261(29.73)	-	334(31.78)	334(31.93)	-
Blood urea nitrogen, n (%)			0.09			<0.01			0.01
≤8 μmol/L	3410(95.04)	896(85.66)	-	1438(92.18)	794(90.43)	-	889(84.50)	896(85.66)	-
＞8 μmol/L	178(4.96)	150(14.34)	-	122(7.82)	84(9.57)	-	163(15.50)	150(14.34)	-
Serum albumin, n (%)*			0.19			0.02			0.02
Normal	2949(82.19)	661(63.19)	-	1152(73.85)	608(69.25)	-	681(64.71)	661(63.19)	-
Abnormal	639(17.81)	385(36.81)	-	408(26.15)	270(30.75)	-	371(35.29)	385(36.81)	-
Serum globulin, n (%)*			0.06			0.01			0.02
Normal	2361(65.80)	621(59.37)	-	916(58.72)	528(60.14)	-	605(57.46)	621(59.37)	-
Abnormal	1227(34.20)	425(40.63)	-	644(41.28)	350(39.86)	-	448(42.54)	425(40.63)	-
Total bilirubin, n (%)			0.02			<0.01			0.01
＞19μmol/L	3385(94.34)	970(92.73)	-	1469(94.17)	825(93.96)	-	988(93.85)	970(92.73)	-

Table S4 Baseline characteristics and SMD of patients after propensity score matching and propensity score weighting.

Characteristic	Primary data			After propensity score matching			After propensity score weighting		
	With use of PPIs	Without use of PPIs	SMD	With use of PPIs	Without use of PPIs	SMD	With use of PPIs	Without use of PPIs	SMD
⩽19μmol/L	203(5.66)	76(7.27)	-	91(5.83)	53(6.04)	-	65(6.15)	76(7.27)	-
Alkaline phosphatase, n (%)			0.04			0.02			<0.01
＞100 IU/L	3215(89.60)	892(85.28)	-	1394(89.36)	766(87.24)	-	894(84.92)	892(85.28)	-
⩽100 IU/L	373(10.40)	154(14.72)	-	166(10.64)	112(12.76)	-	159(15.08)	154(14.72)	-
Oxyhemoglobin saturation, n (%)			0.02			<0.01			0.03
⩾90%	3563(99.30)	1018(97.32)	-	1541(98.78)	865(98.52)	-	995(94.53)	1018(97.32)	-
＜90%	25(0.70)	28(2.68)	-	19(1.22)	13(1.48)	-	58(5.47)	28(2.68)	-
History of hypertension, n (%)	1091(30.41)	386(36.90)	0.06	521(33.40)	301(34.28)	<0.01	365(34.66)	386(36.90)	0.02
History of diabetes mellitus, n (%)	451(12.57)	177(16.92)	0.04	249(15.96)	137(15.60)	<0.01	197(18.76)	177(16.92)	0.02
History of malignant tumor, n (%)	30(0.84)	12(1.15)	<0.01	16(1.03)	8(0.91)	<0.01	10(0.92)	12(1.15)	<0.01
History of stroke, n (%)	95(2.65)	53(5.07)	0.02	64(4.10)	38(4.33)	<0.01	70(6.69)	53(5.07)	0.02
History of COPD, n (%)	25(0.70)	23(2.20)	0.02	17(1.09)	12(1.37)	<0.01	18(1.74)	23(2.20)	<0.01
History of coronary heart disease, n (%)	165(4.60)	116(11.09)	0.06	128(8.21)	82(9.34)	<0.01	122(11.59)	116(11.09)	<0.01
History of digestive system disease, n (%)	278(7.75)	181(17.30)	0.10	206(13.21)	132(15.03)	<0.01	181(17.16)	181(17.30)	<0.01
Antiviral treatment, n (%)	2032(56.63)	701(67.02)	0.10	1002(64.23)	565(64.35)	0.01	709(67.40)	701(67.02)	<0.01
Antibiotic therapy, n (%)	1056(29.43)	558(53.35)	0.24	639(40.96)	404(46.01)	0.01	558(53.03)	558(53.35)	<0.01
Antifungal therapy, n (%)	6(0.17)	34(3.25)	0.03	6(0.38)	6(0.68)	<0.01	33(3.09)	34(3.25)	<0.01
Immunotherapy, n (%)	499(13.91)	250(23.90)	0.10	268(17.18)	171(19.48)	<0.01	240(22.85)	250(23.90)	0.01

Table S4 Baseline characteristics and SMD of patients after propensity score matching and propensity score weighting.

Characteristic	Primary data			After propensity score matching			After propensity score weighting		
	With use of PPIs	Without use of PPIs	SMD	With use of PPIs	Without use of PPIs	SMD	With use of PPIs	Without use of PPIs	SMD
Hormonotherapy, n (%)	231(6.44)	315(30.11)	0.24	215(13.78)	167(19.02)	<0.01	329(31.29)	315(30.11)	0.01
Transfer to ICU, n (%)	17(0.47)	50(4.78)	<0.01	15(0.96)	11(1.25)	<0.01	65(6.18)	50(4.78)	<0.01

Supplementary Table 5 Outcomes of the univariate and multivariate logistic regression with data without missing values imputation.

Supplementary Table 5 Outcomes of the univariate and multivariate logistic regression with data without missing values imputation.

Characteristic	Univariate Logistic Regression		Model 5	
	OR (95%CI)	P value	OR (95%CI)	P value
PPIs usage	19.61(10.54-36.49)	<0.0001	6.35(2.61-15.43)	<0.0001
Age group, years				
18-40	Ref			
41-65	4.23(0.56-31.88)	0.1612		
⩾65	24.24(3.35-175.45)	0.0016		
Sex				
Female	Ref			
Male	2.41(1.48-3.93)	0.0004		
Medical insurance				
Medical insurance for urban	Ref			
New rural cooperative medical insurance	0.74(0.10-5.52)	0.7732		
Free medical treatment	0.61(0.27-1.38)	0.2362		
Self-pay	0.99(0.44-2.24)	0.9765		
Other	1.06(0.64-1.77)	0.8200		
Severity of illness				

Supplementary Table 5 Outcomes of the univariate and multivariate logistic regression with data without missing values imputation.

Characteristic	Univariate Logistic Regression		Model 5	
	OR (95%CI)	P value	OR (95%CI)	P value
Minor	Ref			
Serious	16.66(9.54-29.08)	<0.0001		
Admission temperature				
≤37.0℃	Ref			
＞37.0℃	5.16(2.99-8.91)	<0.0001		
Systolic blood pressure				
Normal	Ref			
Abnormal	1.72(1.07-2.77)	0.0253		
Diastolic blood pressure				
Normal	Ref			
Abnormal	0.94(0.53-1.66)	0.8245		
Pulse rate				
Normal	Ref			
Abnormal	0.79(0.43-1.45)	0.4446		
White blood cell count				
≤9.5×10^9/L	Ref			
＞9.5×10^9/L	11.72(7.25-18.95)	<0.0001		
Red blood cell count*				
Normal	Ref			
Lower	4.32(2.56-7.29)	<0.0001		

Supplementary Table 5 Outcomes of the univariate and multivariate logistic regression with data without missing values imputation.

Characteristic	Univariate Logistic Regression		Model 5	
	OR (95%CI)	P value	OR (95%CI)	P value
Higher	3.37(0.44-25.83)	0.2426		
Mononuclear leucocyte count				
＞0.6×10^9/L	Ref			
⩽0.6×10^9/L	3.58(1.61-7.96)	0.0018		
Neutrophil count				
⩽7×10^9/L	Ref			
＞7×10^9/L	18.06(11.28-28.91)	<0.0001		
Lymphocyte count				
⩽4×10^9/L	Ref			
＞4×10^9/L	3.82(0.50-29.40)	0.1985		
Thrombocyte count				
⩾100×10^9/L	Ref		Ref	
＜100×10^9/L	24.97(14.81-42.07)	<0.0001	10.08(4.44-22.88)	<0.0001
Glutamic-pyruvic transaminase**				
Normal	Ref			
Abnormal	1.86(1.15-3.02)	0.0117		
Glutamic-oxalacetic transaminase				
Normal	Ref			

Supplementary Table 5 Outcomes of the univariate and multivariate logistic regression with data without missing values imputation.

Characteristic	Univariate Logistic Regression		Model 5	
	OR (95%CI)	P value	OR (95%CI)	P value
Abnormal	6.70(4.03-11.15)	<0.0001		
Serum creatinine				
≤73 μmol/L	Ref			
＞73 μmol/L	3.26(2.04-5.20)	<0.0001		
Blood urea nitrogen				
≤8 μmol/L	Ref		Ref	
＞8 μmol/L	18.88(11.71-30.42)	<0.0001	4.14(2.13-8.06)	<0.0001
Serum albumin				
Normal	Ref			
Abnormal	14.54(8.21-25.76)	<0.0001		
Serum globulin				
Normal	Ref			
Abnormal	0.86(0.53-1.40)	0.5472		
Total bilirubin				
＞19μmol/L	Ref			
≤19μmol/L	4.67(2.67-8.15)	<0.0001		
Alkaline phosphatase				
＞100 IU/L	Ref			
≤100 IU/L	4.31(2.64-7.01)	<0.0001		
Oxyhemoglobin saturation				

Supplementary Table 5 Outcomes of the univariate and multivariate logistic regression with data without missing values imputation.

Characteristic	Univariate Logistic Regression		Model 5	
	OR (95%CI)	P value	OR (95%CI)	P value
⩾90%	Ref		Ref	
﹤90%	30.41(15.62-59.20)	<0.0001	4.24(1.51-11.86)	0.0060
History of hypertension	2.19(1.39-3.46)	0.0007		
History of diabetes mellitus	2.41(1.43-4.04)	0.0009		
History of malignant tumor	5.29(1.58-17.70)	0.0068		
History of stroke	5.70(2.93-11.09)	<0.0001	7.32(2.60-20.61)	0.0002
History of COPD	7.80(2.97-20.45)	<0.0001		
History of coronary heart disease	5.21(3.05-8.89)	<0.0001		
History of digestive system disease	2.06(1.14-3.72)	0.0160		
Antiviral treatment	2.21(1.30-3.76)	0.0035		
Antibiotic therapy	145.83(20.25-1049.93	<0.0001	30.92(3.88-246.30)	0.0012
Antifungal therapy	48.63(22.66-104.37)	<0.0001		
Immunotherapy	3.49(2.18-5.56)	<0.0001		
Hormonotherapy	35.53(20.02-63.08)	<0.0001	5.38(2.50-11.60)	<0.0001
Transfer to ICU	81.53(44.60-149.04)	<0.0001	13.54(5.87-31.23)	<0.0001

Figure S1

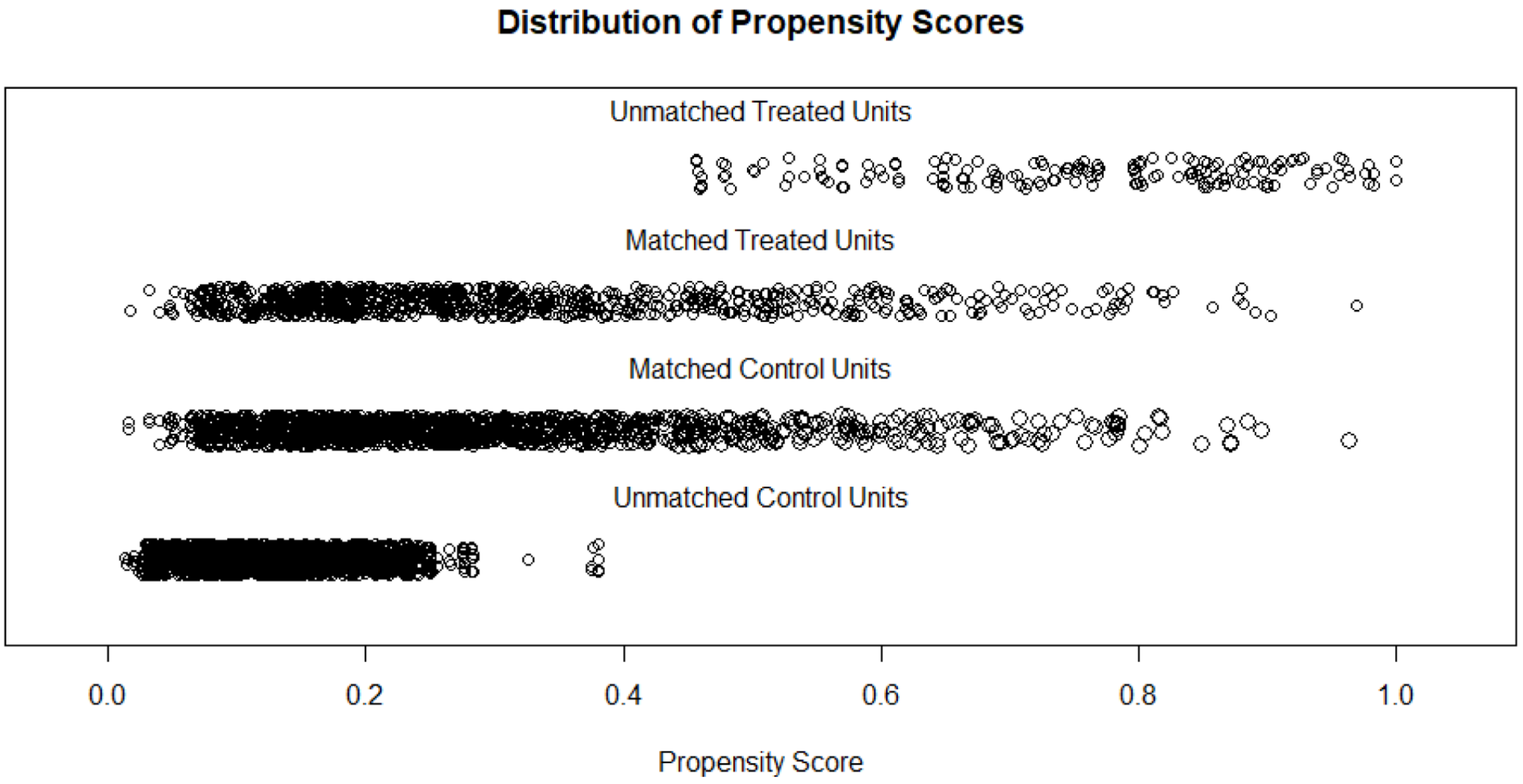
Distribution of Propensity Scores
Unmatched Treated Units
Matched Treated Units
Matched Control Units
Unmatched Control Units
0.0
0.2
0.4
0.6
0.8
1.0
Propensity Score

Figure S2.

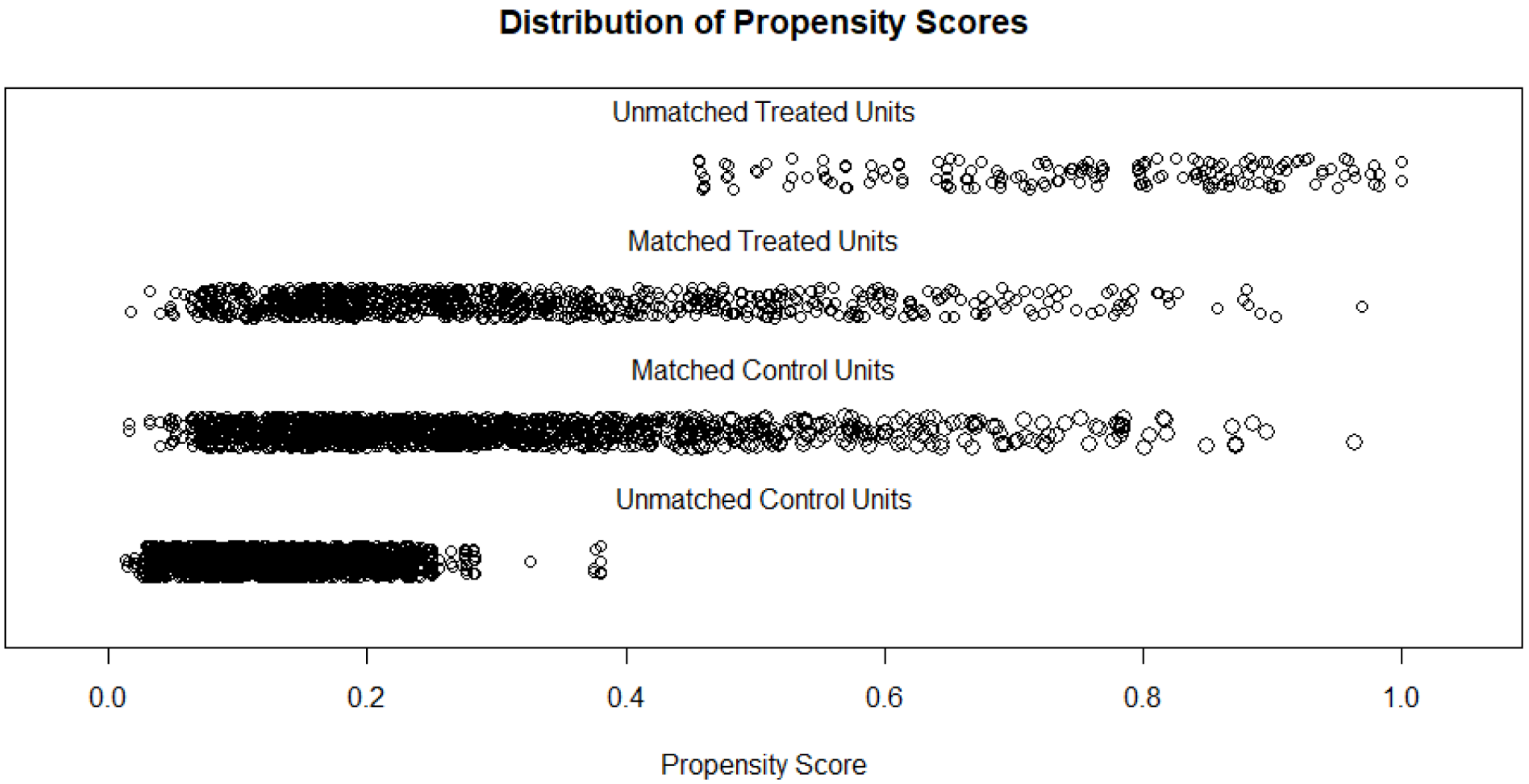
Distribution of Propensity Scores
Unmatched Treated Units
Matched Treated Units
Matched Control Units
Unmatched Control Units
0.0
0.2
0.4
0.6
0.8
1.0
Propensity Score
